# ‘Schizophrenia is a dirty word’: service users’ experiences of receiving a diagnosis of schizophrenia

**DOI:** 10.1192/pb.bp.113.045179

**Published:** 2014-08

**Authors:** Lorna Howe, Anna Tickle, Ian Brown

**Affiliations:** 1 Nottingham University, UK; 2 Nottinghamshire Healthcare NHS Trust, UK

## Abstract

**Aims and method** To explore service users’ experiences of receiving a diagnosis of schizophrenia and the stigma associated with the diagnostic label. Seven participants were interviewed about their perceptions of these experiences. Interviews were analysed using interpretative phenomenological analysis.

**Results** Five superordinate themes resulted from the analysis: (1) avoidance of the diagnosis of schizophrenia; (2) stigma and diagnostic labels; (3) lack of understanding of schizophrenia; (4) managing stigma to maintain normality; (5) being ‘schizophrenic’. These, together with their subthemes, highlighted avoidance of the term schizophrenia by participants and use of alternative terms by professionals, which limited opportunities for understanding the label and challenging associated stigma. Participants strived to maintain normality despite potential stigma.

**Clinical implications** There is a need to address the process of giving a diagnosis as a phenomenon of consequence within its own terms. Implications relate to how professionals deliver and discuss the diagnosis of schizophrenia.

The diagnosis of schizophrenia may be contradictory: as a means of access and explanation *v*. a source of labelling and social exclusion.^1,2^ Evidence suggests it is an extremely powerful diagnosis that can have a devastating impact on a person’s self-identity.^3^ Stigma can lead individuals to internalise negative attitudes, resulting in low self-esteem and social isolation.^4^ Alternatively, individuals may resist stigma and have a strong sense of group identity and empowerment.^5^ Incorporating the ‘schizophrenic’ identity into a new self-concept may be part of the recovery process.^6^ However, the importance of this experience remains largely silent within psychiatry and mental health services.^7^

Research has neglected perceptions of diagnosed individuals about the experience, thus failing to substantiate diagnosis as a phenomenon of consequence.^8^ The current research aimed to contribute to existing literature and inform the practice of giving the diagnosis of schizophrenia by exploring individuals’ subjective experiences of receiving a diagnosis of schizophrenia and stigma relating to the diagnostic label.

## Method

### Participants

Seven participants diagnosed with schizophrenia were recruited from a community mental health team. Three were male, four female. All were White British and aged 29-60 years (mean 44 years). All had received a diagnosis of schizophrenia between 6 and 17 years earlier (mean 12 years).

### Procedure

Ethical approval was received from a National Health Service (NHS) research ethics committee and an NHS trust. Participants gave informed consent prior to an audio-recorded semi-structured interview lasting between 40 and 90 min, conducted by one of the authors (L.H.). The interview schedule was developed in consultation with colleagues familiar with interpretative phenomenological analysis (IPA) and working with individuals experiencing psychosis.

### Analysis

Data were analysed using IPA procedures,^9^ which explore how people make sense of experiences. All interviews were transcribed verbatim: each transcript was read and re-read with initial notes made regarding meanings and interpretations. Emergent themes were developed to emphasise patterns and connections across exploratory comments. The themes were then connected to develop ‘clustered themes’. Patterns across participants were explored to develop ‘superordinate’ and ‘subordinate’ themes for the entire data-set. Constant re-examination of data ensured the themes remained related to the primary source. Themes were not chosen purely on their prevalence, but also on the depth of data and how they illuminated other themes. Researcher self-reflection was used to maintain awareness of assumptions and their potential impact on analysis, in line with a critical realist epistemological stance.

## Results

**Fig 1:**
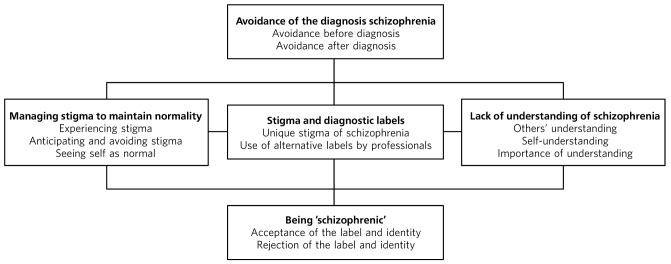
Illustration of the interconnections between themes.

Five interconnected superordinate themes, with two or three subordinate themes each, were drawn from the analysis (Fig. 1). The terms reflect those used by participants (e.g. ‘schizophrenic’), while acknowledging potential difficulties associated with such expressions. Pseudonyms are used with the consent of the participants.

### Theme one: avoidance of the diagnosis of schizophrenia

Participants described avoidance of being diagnosed with schizophrenia and of the term ‘schizophrenia’, which had a significant impact on help-seeking behaviour. Three participants self-identified their symptoms as schizophrenia but avoided seeking help to evade being diagnosed due to fearing the label itself. They talked about not wanting to be ‘ill’, wanting to avoid stigma attached to the label, and concerns that others would not understand:

‘I’d read about it and I knew I had it... but I was too scared to tell the doctors what my real symptoms were so they could treat me’ (Carol).

There appeared to be serious clinical implications of this avoidance of the diagnosis of schizophrenia, including unnecessary distress, misdiagnosis and preventing early intervention. All participants reported being misdiagnosed with depression due to actively avoiding being labelled with schizophrenia.

Once diagnosed, participants furthered avoidance of the term schizophrenia by keeping their diagnosis secret from others, including close family, for fear of being treated differently:

‘I’ve got two step-sons... they think I’m a bit weird but they don’t know I’ve got schizophrenia... we get on okay and I don’t think I want to jeopardise or spoil that’ (David).

In contrast, participants’ eagerness to talk about their diagnosis during the interviews seemed indicative of their desire to talk about it within a safe context. This need seemed largely unmet by professionals and members of participants’ social networks.

### Theme two: stigma and diagnostic labels

Participants described avoidance of telling people about their diagnosis because of its ‘unique’ stigma, which seemed more shameful and hurtful than stigma relating to ‘mental illness’ and other diagnoses:

‘You can tell anybody you’ve got bipolar [disorder], it used to be called manic depression, they just say “oh you’re just depressed”... I think schizophrenia is a unique thing on its own, because it’s always associated with violence, which breaks my heart. It’s always associated with violence’ (Carol).

Participants raised the role played by the media in reinforcing such stigmatisation:

‘It’s really bad information... because everything you watch on television, if there’s [a murder] and that the person has always suffered from [schizophrenia] for a number of years, they always seem to say that’ (Janet).

Participants were specifically asked to describe the experience of receiving a diagnosis of schizophrenia. Responses to this were mostly unclear and reflected uncertainty about the time of diagnosis:

‘I don’t know if I was diagnosed with depression before schizophrenia or not... God I don’t know... I don’t know how they diagnosed me’ (Paul).

Professionals appeared to use alternative, seemingly more acceptable terms such as ‘psychosis’, leading one of the participants to discover his diagnosis in an unexpected way:

‘Nobody really explained to me properly that I’d got schizophrenia... I had one of those [care programmeapproach reviews] and with the paperwork that got mailed through... I noticed it on there... for years I’ve been told that it was psychosis’ (Ben).

When talking about whether there is a difference between psychosis and schizophrenia, he said:

‘I think I’ve dealt quite well with the fact that I’m mentally ill and I sometimes tell people that I suffer from illness. But I think if I was to say to them that I suffer from schizophrenia I don’t think I’d particularly like that. I think there is a kind of stigma attached to it’, later adding ‘It just seems like a word that makes you seem more unwell’ (Ben).

One person noticed that, even after diagnosis, professionals tended to use alternative terms to schizophrenia:

‘People are always afraid of saying that word to me and they’re always saying something else... My [community psychiatric nurse] was too afraid to say it... because it is a dirty word to even say that word schizophrenia. It’s like saying f^*^off or something’ (Carol).

The description of the word ‘schizophrenia’ as distinct from the experience was deemed indicative of stigma being attached specifically to the diagnostic label, suggesting that alternative labels may be perceived to reduce stigma.

### Theme three: lack of understanding of schizophrenia

Participants described how avoidance of the term schizophrenia inhibited understanding more about it. Most perceived a lack of knowledge and understanding from both professionals and family members. A stark contrast was indicated between professionals’ biological understanding and alternative views potentially held by others:

‘I feel doctors sit back and they don’t know... they might give you a diagnosis of schizophrenia... they think it’s some kind of chemical imbalance in the brain... but they can’t explain it’ (David).

‘My mother... all she said was “I told you, it’s because you’re psychic, nothing to do with you being schizophrenic... you’re just psychic” ‘ (Janet).

Professionals’ focus on biological causes led most participants to liken schizophrenia to an inherent physical illness, requiring long-term medical treatment. This conceptualisation seemed to limit hope for recovery and encourage individuals to become passive recipients of care, concluding schizophrenia would always be part of their lives:

‘I think this illness is ongoing for me, I don’t think that I’m going to... miraculously get well from it, so I just go for an injection once a fortnight’ (Helen).

Most participants wanted to develop their understanding of schizophrenia and indicated this could aid acceptance of the diagnosis:

‘Maybe if I’d have had it explained better to me in the beginning I might have accepted it better, and I think that would have helped me... a great deal’ (Janet).

In the absence of discussion of schizophrenia by professionals, one person turned to others with the same diagnosis for information. He highlighted the importance of shared experiences and peer support, but also the potential for services to better support individuals develop their understanding:

‘I’ve been to some seminars... where people who have got schizophrenia have got up on stage and talked about it... I’ve related to some of those things more so than [to] what doctors have said to me’ (David).

### Theme four: managing stigma to maintain normality

All participants reported seeing themselves as ‘normal’ and resisted internalising stigma. This was perceived as difficult in light of experienced and anticipated stigma, which seemed to have an impact on participants’ semblance of normality. Most described personal experiences of stigmatisation from family members and professionals.

‘I’ve had police... not that I’ve done anything wrong, but when it’s someone I’ve been associated with, and they’ve said “You’re not going to bring an axe out to me are you?”. Police! Even police’ (Carol).

Subtle experiences of others’ benevolent ‘over-concern’ and judgements of incompetence and unintelligence also appeared to be common experiences:

‘When the doctor comes here, she always turns to my social worker to ask him. They’re treating me like I’m subnormal because I think they saw me as not being able to understand’ (Janet).

All participants conveyed constant awareness of potential stigma if others learned of their diagnosis. They described trying to maintain normality and protect themselves from stigma by keeping their diagnosis secret and managing others’ perceptions of them.

‘I overcompensate by being extra kind I think... trying to ring people up you know, have a chat... just to feel like they think I’m normal’ (Carol).

These strategies reflected a desire to maintain relationships, but paradoxically prevented relational closeness; most participants described being ‘isolated’ and lacking ‘close friends’.

‘I’ve isolated myself. I have been invited to different things but I decline offers; I don’t want to be in a position where people get to know me. Nobody likes rejection, so I just don’t put myself in that position’ (Janet).

Most participants appeared to condemn stigma associated with schizophrenia and perceived themselves as ‘normal’:

‘It does bother me because I am pretty normal in most ways... to have people treat me like that, you want to say “Look, I’m not that bad” ‘ (Ben).

This was interpreted as explicit attempts to delineate others’ expectations from their own expectations and resist internalising stigma. However, this strategy had not been successful for one person, who highlighted a conflict in managing her self-identity:

‘I was frightened of my own self because I’d only associated schizophrenia with people who hurt other people. I kept thinking, oh my God, I’m going to end up like that, I’m going to end up a killer, so I was terrified of going out. I personally put the stigma on myself’ (Janet).

### Theme five: being ‘schizophrenic’

Participants described coming to terms with their diagnosis and accepting or rejecting schizophrenia as part of their identity. Most talked about diagnosis as enabling access to treatment, which paved the way to a greater sense of acceptance:

‘It’s nice to get [the diagnosis] because then you get help. I bet there’s people not getting any help because they don’t believe they’ve got [schizophrenia]’ (Paul).

For some, being diagnosed confirmed what they already ‘knew’; this indicated acceptance of the label as a description of their difficulties:

‘It was like a relief in a way that at least they knew now what I already knew, that I’d got this schizophrenia’ (David).

Such acceptance also required disconfirmation of the stigma surrounding schizophrenia, and a sense of group identity and self-identity as being ‘schizophrenic’:

‘If you’d took me when I thought I was alright and put me in the middle of a class full of schizophrenics I’d have been worried. I’d have thought “these could be violent”. But actually having [schizophrenia] myself and knowing it doesn’t really make you violent I can cope... they’re all nice people’ (Paul).

In contrast to other participants, one person rejected the label of schizophrenia and described a lack of group identity:

‘I went to [the mental health charity] Mind and there was supposed to be people there who were schizophrenics, but they were so different from me and their symptoms were different from mine. I didn’t fit into their category at all’ (Janet).

However, this did not mean that the person lacked ‘insight’ or refused the need for treatment:

‘I knew I was quite ill... I did want them to give me something to make me feel a bit better... I know you’ve got to... put a name to a certain condition. I can accept that I’ve got mental health problems, but I will not accept that I’m schizophrenic’ (Janet).

This seemed linked to her fears of the image of schizophrenia, leaving her in the difficult position of needing a diagnosis to access treatment, but wanting to avoid diagnosis due to self-stigma.

## Discussion

Participants were seen to describe constant social and psychological processes involved in avoiding schizophrenia, trying to understand it, managing the stigma surrounding it, and becoming it (i.e. ‘schizophrenic’).

The findings reflect and expand on the literature. Participants described the contradictory nature of being diagnosed with schizophrenia as a means of access *v*. a source of stigma and social exclusion,^1,2^ and reported being subject especially to perceptions of dangerousness and incompetence.^10,11^ The findings also reflect those found elsewhere that the diagnostic label of schizophrenia, in part reflected in media reporting, is associated with greater stigma than other psychiatric diagnoses.^12^ Participants also appeared to experience ‘benevolent stigma’, a reportedly common attitude towards people with other mental health problems but not specifically schizophrenia.^13^ There was an indication that professionals withheld detailed diagnostic information from participants,^7,14^ contributing to their anticipation of negative reactions, which resulted in diagnostic secrecy and decreased social relationships.^12,15,16^

Interestingly, there was a distinct lack of self-stigma in participants’ statements; all but one set concealment in the context of anticipating stigmatising reactions from others, rather than believing themselves to be in some way defective. This contradicts long-standing theories that represent self-stigma as inherent for individuals labelled as ‘mentally ill’.^4^ This finding draws on more recent literature on the apparent paradox of stigma as potentially resulting in a significant loss of self-esteem for some, whereas others may be energised or righteously angry about stigma and resist or denounce it.^17^

An important finding was that avoidance preceded diagnosis; participants hid their experiences to avoid being labelled with schizophrenia, consequently receiving inappropriate treatments. This highlights participants’ determination to evade the specific diagnosis of schizophrenia to protect themselves from stigma at the price of receiving more appropriate treatment. Previous research suggests individuals with longer duration of untreated psychosis have worse clinical outcomes regarding symptom severity and social disadvantage.^18^ Therefore, avoidance must be overcome to promote better outcomes for individuals.

Following diagnosis, professionals may have used alternative labels such as ‘psychosis’ to limit the impact of the unique stigma associated with schizophrenia. However, this led to some participants not knowing their diagnosis and appeared to maintain a lack of understanding of schizophrenia, both stemming from and perpetuating stigma related to the diagnosis.^19^ Consistent with existing literature, participants mostly understood schizophrenia as a biological illness, which reduced hope for change and increased reliance on medication as well as a sense of passivity.^20^ Professionals may need to take the lead and break their ‘conspiracy of silence’ surrounding schizophrenia to allow the public and those with the diagnosis to follow.^7^

Participants’ group identity appeared generally to relate to acceptance of diagnosis and even self-labelling as ‘schizophrenic’, in line with the ‘I am’ nature of the diagnosis.^21^ The present research suggests a dilemma whereby individuals required their diagnosis to access support and gain acknowledgement of their experiences, but struggled with their sense of self and prospect of being ‘schizophrenic’ permanently. As a result, a compromised identity incorporating the old self and the new ‘schizophrenic’ self was needed to manage stigma and maintain normality, while also accepting their diagnosis.

### Limitations

This study relied on retrospective accounts at different time periods following diagnosis, possibly representing different stages of coming to terms with it. Those who received their diagnosis more recently may have had a different experience to those who were diagnosed many years ago, although this was not evident in the results.

### Implications

The saliency of stigma in participants’ lives despite the alleviation of symptoms related to their diagnosis signifies the personal and social burden of being labelled with schizophrenia. It would appear that one response by professionals is to resort to alternative terms; however, this may lead to confusion. Professionals have an important role in addressing stigma associated with schizophrenia beyond psychoeducation and focusing on symptomatology and medication. Clinical practice and anti-stigma campaigns may benefit from focusing on the psychosocial causes of a person’s difficulties, and contextualising and making sense of people’s experiences rather than focusing on a biological approach. Due to the nature of stigma, it is unlikely that service users will bring it directly to the attention of professionals. Therefore, professionals should specifically ask about experiences of stigma, the extent of social networks, self-image and ‘new’ identity post-diagnosis.^22^

Further investigations focusing on the issues raised in the present study may greatly benefit clinical practice with individuals deemed to meet the diagnostic criteria for schizophrenia, including the use and communication of the diagnosis and the associated stigma which may have an impact on service users’ lives.
